# Supplementary material for: CpBBX19, a B-Box Transcription Factor Gene of Chimonanthus praecox, Improves Salt and Drought Tolerance in Arabidopsis
Source: Genes (Basel). 2021 Sep 21;12(9):1456. doi: 10.3390/genes12091456 (PMC8465485; doi:10.3390/genes12091456)
Supplement: Supplementary file 1 [file genes-12-01456-s001.zip › genes-1366083-supplementary.pdf]

Table S1. List of primers.

Primer name	Primer sequences (5′-3′)
CpBBX19-F	GAAGGGAAATGAAAGGTGGG
CpBBX19-R	CTTCTTTAAGCCGCCAATAGTC
CpBBX19-F1	ATGCGAACCCTCTGCGACGTTTGC
CpBBX19-R2	TTACTTCTCACACACACCTTTG
pGWB551-CpBBX19-F	GGGGACAAGTTTGTACAAAAAAGCAGGCT[1]<br>GAAGGGAAATGAAAGGTGGG
pGWB551-CpBBX19-R	GGGGACCACTTTGTACAAGAAAGCTGGGT[2]<br>CTTCTTTAAGCCGCCAATAGTC
RT-*CpActin*-F	AGGCTAAGATTCAAGACAAGG
RT-*CpActin*-R	TTGGTCGCAGCTGATTGCTGTG
RT-*AtActin*-F	CTTCGTCTTCCACTTCAG
RT-*AtActin*-R	ATCATACCAGTCTCAACAC
RT-*CpBBX19*-F	AACCTAGTTCTTGTGAAGACTTGGC
RT-*CpBBX19*-R	CATCCATGTTAGCATCATTAGTTGG
SP1	CTCAACTGGGTCCATTGGTTGCAAG
SP2	TAAAAGAAAGCAGGTGCATTTTCACATATG

[1] stands for *att*B1, [2] stands for *att*B2

Table S2. Analysis of cis-acting elements of promoters. The amounts are the sum of Cis-elements in sense and antisense strand.

No.	Name of elements	Amount	Sequence	function
1	TATA-box	1	TATA	core promoter element
2	CAAT-box	1	CAAAT/CAAT/CCAAT	common cis-acting element in promoter and enhancer regions
3	ABRE	3	GCAACGTGTC/ACGTG/CACGTG	cis-acting element involved in the abscisic acid responsiveness
4	ARE	1	AAACCA	cis-acting regulatory element essential for the anaerobic induction
5	CGTCA-motif	2	CGTCA	cis-acting regulatory element involved in the MeJA-responsiveness
6	MBS	1	CAACTG	MYB binding site involved in drought-inducibility
7	MYB	2	CAACCA	
8	MYC	4	CATTTG	
9	MYb	1	CAACTG	
10	MYc	1	TCTCTTA	
11	TC-rich	1	GTTTTCTTAC	cis-acting element involved in defense and stress

				responsiveness
12	TCA-element	1	CCATCTTTTT	cis-acting element involved in salicylic acid responsiveness
13	TGACG-motif	2	TGACG	cis-acting regulatory element involved in the MeJA-responsiveness

Table S3. Names and accession numbers used for phylogenetic analysis

Name	Species	Accession number
AtBBX1	*Arabidopsis thaliana*	AT5G15840
AtBBX2	*Arabidopsis thaliana*	AT5G15850
AtBBX3	*Arabidopsis thaliana*	AT3G02380
AtBBX4	*Arabidopsis thaliana*	AT2G24790
AtBBX5	*Arabidopsis thaliana*	AT5G24930
AtBBX6	*Arabidopsis thaliana*	AT5G57660
AtBBX7	*Arabidopsis thaliana*	AT3G07650
AtBBX8	*Arabidopsis thaliana*	AT5G48250
AtBBX9	*Arabidopsis thaliana*	AT4G15250
AtBBX10	*Arabidopsis thaliana*	AT3G21880
AtBBX11	*Arabidopsis thaliana*	AT2G47890
AtBBX12	*Arabidopsis thaliana*	AT2G33500
AtBBX13	*Arabidopsis thaliana*	AT1G28050
AtBBX14	*Arabidopsis thaliana*	AT1G68520
AtBBX15	*Arabidopsis thaliana*	AT1G25440
AtBBX16	*Arabidopsis thaliana*	AT1G73870
AtBBX17	*Arabidopsis thaliana*	AT1G49130
AtBBX18	*Arabidopsis thaliana*	AT2G21320
AtBBX19	*Arabidopsis thaliana*	AT4G38960
AtBBX20	*Arabidopsis thaliana*	AT4G39070
AtBBX21	*Arabidopsis thaliana*	AT1G75540
AtBBX22	*Arabidopsis thaliana*	AT1G78600
AtBBX23	*Arabidopsis thaliana*	AT4G10240
AtBBX24	*Arabidopsis thaliana*	AT1G06040
AtBBX25	*Arabidopsis thaliana*	AT2G31380
AtBBX26	*Arabidopsis thaliana*	AT1G60250
AtBBX27	*Arabidopsis thaliana*	AT1G68190
AtBBX28	*Arabidopsis thaliana*	AT4G27310
AtBBX29	*Arabidopsis thaliana*	AT5G54470
AtBBX30	*Arabidopsis thaliana*	AT4G15248
AtBBX31	*Arabidopsis thaliana*	AT3G21890
AtBBX32	*Arabidopsis thaliana*	AT3G21150

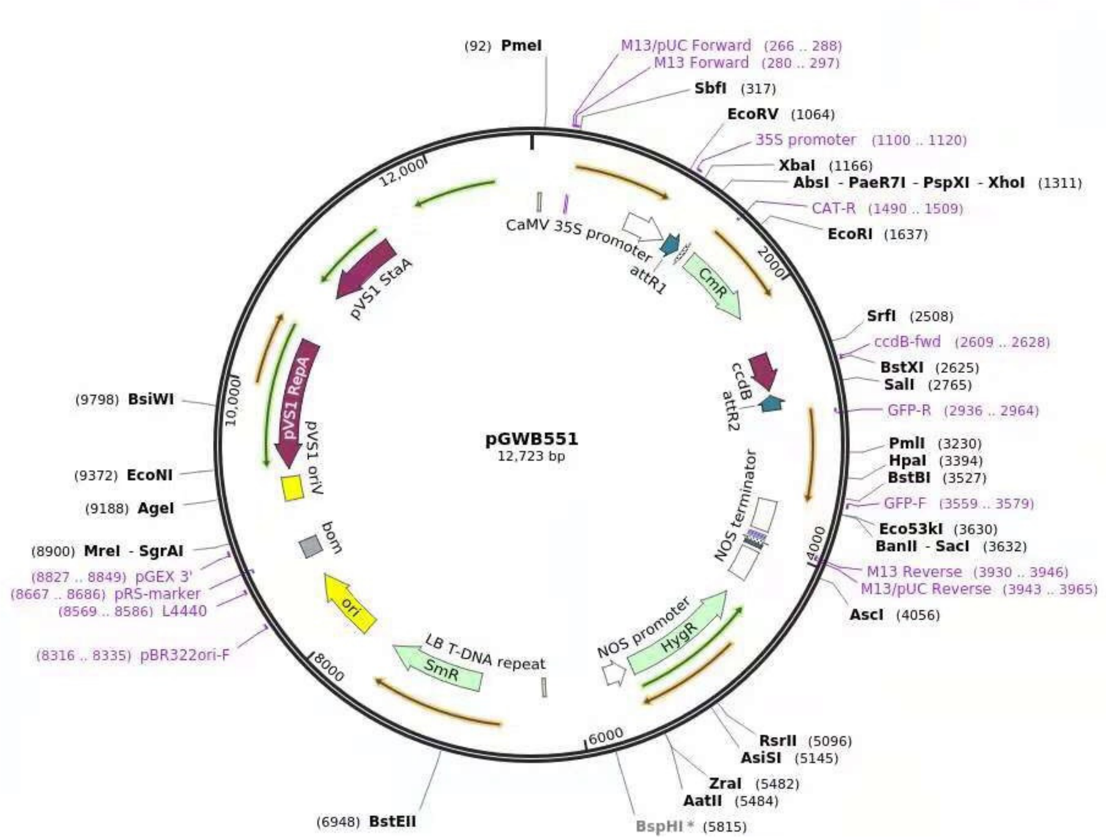

Figure S1. The simple map of the pGWB551 vector.

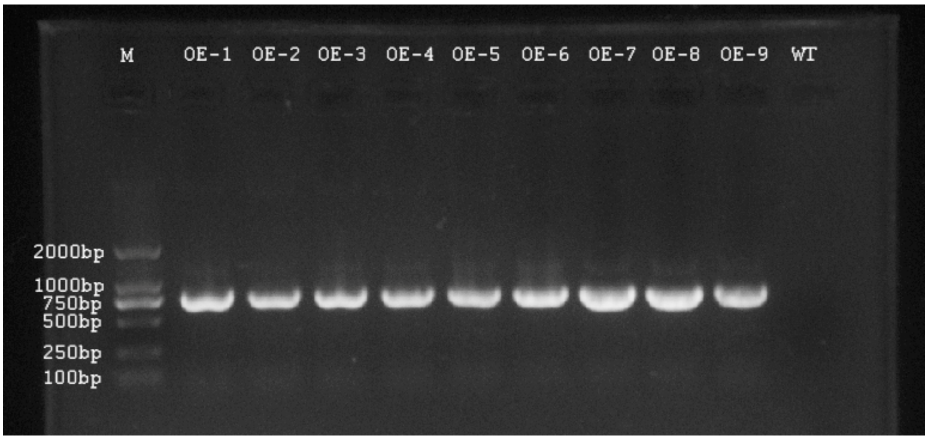

Figure S2. PCR Detection of *CpBBX19* overexpression *Arabidopsis*. OE-1-9: *CpBBX19* transgenic *Arabidopsis*; WT: wild-type Arabidopsis. M: DNA Maker DL2000.
